# A split protease-*E. coli* ClpXP system quantifies protein–protein interactions in *Escherichia coli* cells

**DOI:** 10.1038/s42003-021-02374-w

**Published:** 2021-07-06

**Authors:** Shengchen Wang, Faying Zhang, Meng Mei, Ting Wang, Yueli Yun, Shihui Yang, Guimin Zhang, Li Yi

**Affiliations:** State Key Laboratory of Biocatalysis and Enzyme Engineering, School of Life Sciences, Hubei University, Hubei, China

**Keywords:** Molecular engineering, Microbiology techniques

## Abstract

Characterizing protein–protein interactions (PPIs) is an effective method to help explore protein function. Here, through integrating a newly identified split human Rhinovirus 3 C (HRV 3 C) protease, super-folder GFP (sfGFP), and ClpXP-SsrA protein degradation machinery, we developed a fluorescence-assisted single-cell methodology (split protease-*E. coli* ClpXP (SPEC)) to explore protein–protein interactions for both eukaryotic and prokaryotic species in *E. coli* cells. We firstly identified a highly efficient split HRV 3 C protease with high re-assembly ability and then incorporated it into the SPEC method. The SPEC method could convert the cellular protein-protein interaction to quantitative fluorescence signals through a split HRV 3 C protease-mediated proteolytic reaction with high efficiency and broad temperature adaptability. Using SPEC method, we explored the interactions among effectors of representative type I-E and I-F CRISPR/Cas complexes, which combining with subsequent studies of Cas3 mutations conferred further understanding of the functions and structures of CRISPR/Cas complexes.

## Abstract (web summary)

Wang et al. developed a fluorescence-assisted single-cell methodology (split protease-*E. coli* ClpXP (SPEC)) to characterise protein-protein interactions for both eukaryotic and prokaryotic species in *E. coli* cells. Their method can quantify these interactions with high sensitivity, easy manipulation, and broad temperature adaptability

## Introduction

Proteins serve essential functions in nearly all biological processes in living organisms. It has been revealed that over 80% of proteins exist in complexes^1^, suggesting that protein is normally not carrying out its function alone. Therefore, characterizing the protein–protein interactions (PPIs) could help reveal the functions of the proteins, and also prompt the understanding of biological processes which they are involved in. Many methods have been developed for characterizing the PPIs, including bacteria/yeast two-hybrid (B2H/Y2H)^2,3^, affinity-based techniques^4,5^, quantitative proteomics^6^, and split protein complementation assays^7^. For example, Y2H has developed 30 years ago, which has been used to confirm suspected interactions, identify protein–drug interaction, and discover inhibitors of the PPIs^8^. In addition, quantitative proteomics is a newly developed method, which was recently used to map receptor-ligand interactions in the activation mechanism of class B G-protein-coupled receptors (GPCRs) upon interaction with peptide hormones in signal-transduction pathways of mammalian cells and elucidate the protein interactome in chromatin biology^9^.

Besides these methods, split-protein assays, also termed protein fragment complementation assays (PCAs), are another set of methods for characterizing PPIs, including split-GFP^10^, split-luciferase^11^, split-ubiquitin^12^, and split-TEV protease^13^ et al. PCAs have been widely applied in both prokaryotic and eukaryotic species to characterize transient and irreversible PPIs, and manipulate signal pathways, which consist of modular elements to prompt the system flexibility and functionality under in vivo environment. For example, split TEV protease assay was used to monitor the neuregulin-induced ErbB2/ErbB4 receptor tyrosine kinase heterodimerization^13^, and GPCR activities caused by drug-induced changes^14^. Although PCAs has been proven to be a robust method for explore PPIs, designing functional split proteins is a very challenging task, which needs to maintain the stability as well as the functionality of the split proteins to ensure the recovered biological activity of the reassembled proteins^15^. Many split proteins have been identified so far, among which split protease has the advantages of signal amplification and clean background because of its high catalytic activity and substrate specificity. Small amount of reassembled proteases could cleave large amount of its substrates, thus generating enhanced signals to increase the systematic sensitivity.

*E. coli* is the dominant model species in biological research due to its easy manipulation and clear genetic background. Recently, engineered *E. coli* was also used for eukaryotic protein studies^16,17^, and the good adaption to low temperature further favors its use in academia and industry. Therefore, it is attractive to develop a sensitive PCA for characterizing PPIs in *E. coli* within a broad temperature range. Through combining the ClpXP-SsrA protein degradation machinery^18^, sfGFP fluorescence reporter, we developed a split human Rhinovirus (HRV) 3 C protease mediated PCA in *E. coli* cells, termed as Split Protease-*E. coli* ClpXP (SPEC) system. Our studies showed that splitting at K82 position of HRV 3 C protease could generate a high effective split HRV 3 C protease, and its incorporation to the SPEC system can make it characterize PPIs in *E. coli* quantitatively with high sensitivity, easy manipulation, and broad temperature adaptability from 18 to 37 °C. Using SPEC method, we further analyzed the interactions of effects of type I-E and I-F CRISPR/Cas to understand its possible complex structure and functions.

## Results

### Development of SPEC system

**Fig. 1:**
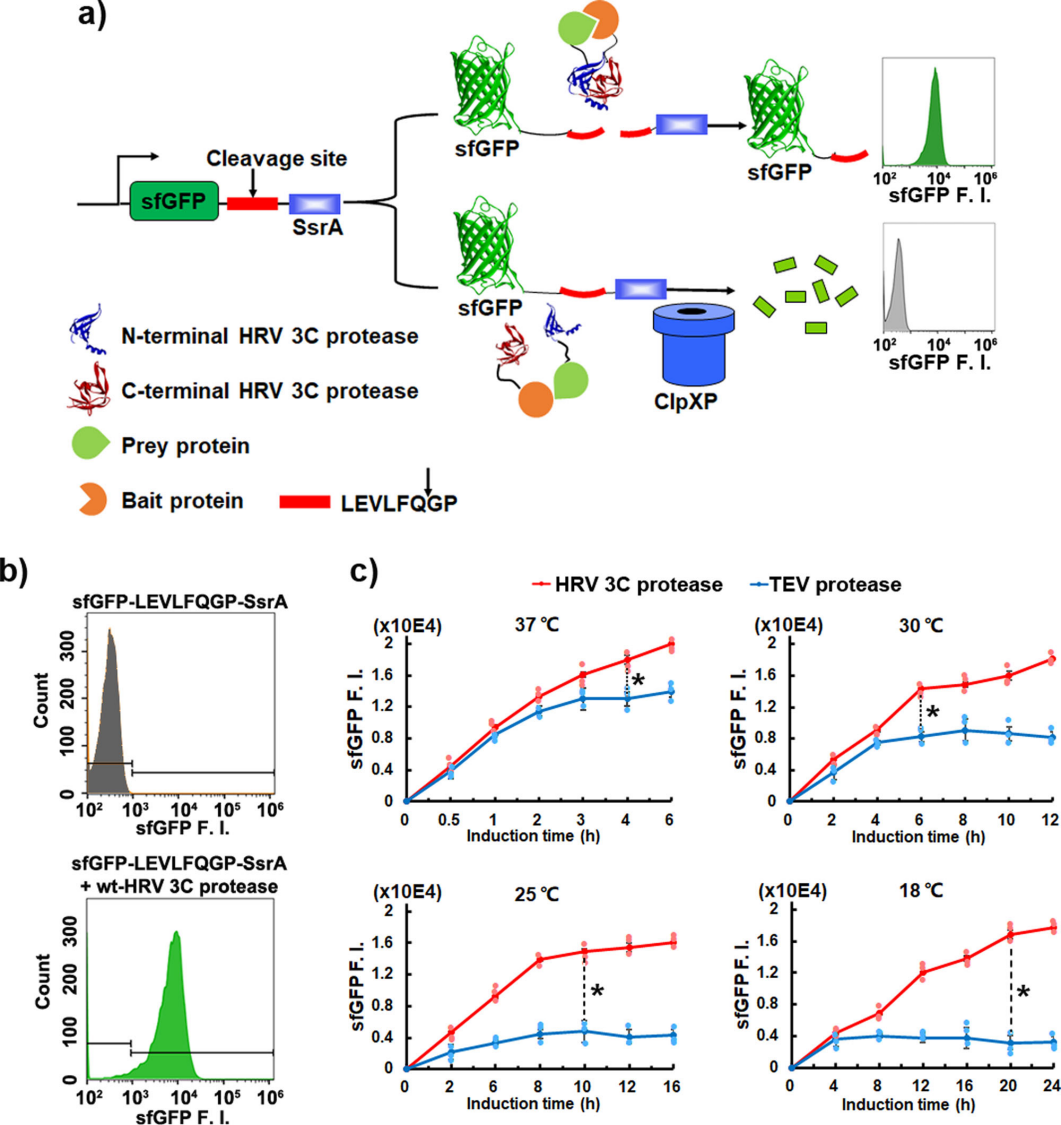
Development of the concept of SPEC system. **a** Schematic diagram of Split Protease-*E. coli* ClpXP (SPEC) system. sfGFP is fused with an HRV 3 C protease cleavage sequence (LEVLFQ↓GP) at its C-terminus followed by a SsrA sequence (AANDENYALAA), forming a sfGFP-LEVLFQGP-SsrA cassette. The prey protein and bait protein are fused to the N-terminal and C-terminal split-HRV 3 C proteases, respectively. Through ClpXP-SsrA degradation pathway, highly expressed sfGFP-LEVLFQGP-SsrA cassette is fast degraded in *E. coli* cells, thus very low green fluorescence is detected. Nevertheless, when prey protein interacts with bait protein, the N-terminal split and C-terminal split-HRV 3 C proteases will re-assemble to a functional HRV 3 C protease, which will cleave off the SsrA peptide from the sfGFP-LEVLFQ↓GP-SsrA cassette, leading to an accumulation of sfGFP for increased fluorescence intensity. **b** Validation of the ClpXP-SsrA protein degradation machinery mediated sfGFP degradation in *E. coli*. The total cellular sfGFP fluorescence intensity (sfGFP F. I.) of cells bearing different plasmids were quantitated by flow cytometry (detected with FITC channel, 525/40 nm BP). The experiments were performed under 25 °C with 8 h induction. Upper panel: cells bearing pSPEC-VA vector, Lower panel: cells bearing both pSPEC-VA and pSPEC-VB vectors (Supplementary Table 1, Supplementary Fig. 1). **c** Comparing TEV protease (blue line, pSPEC-T, Supplementary Table 1) with HRV 3 C protease (red line, pSPEC-VB, Supplementary Table 1) in SPEC system at different temperatures of 37 °C, 30 °C, 25 °C, and 18 °C, respectively. All the vector information can be found in Supplementary Table 1. Data are presented as mean ± SEM (*n* = 3 independent experiments) with Student’s t test being performed, ^#^*P* > 0.05, *P ≤ 0.05.

The SPEC system consists of two sub-parts, the ClpXP-SsrA protein degradation machinery mediated sfGFP fluorescence reporting sub-system and the split-HRV 3 C protease-mediated protein interacting sub-system (Fig. 1a). In *E. coli*, ClpXP protease can recognize the SsrA sequence (AANDENYALAA), thus causing the degradation of SsrA-tagged protein in cells^18^. When the prey protein and bait protein do not interact with each other, sfGFP-SsrA is degraded through the ClpXP-SsrA mediated protein degradation pathway accompanied with a low cellular sfGFP fluorescence intensity. However, when the prey protein and bait protein interact with each other, their fused N-terminal and C-terminal split HRV 3 C proteases can re-assemble to a functional HRV 3 C protease, cleaving the SsrA peptide off sfGFP-SsrA to cause an increased sfGFP fluorescence intensity.

We first validated the principle of SPEC system in *E. coli* BL21(DE3) by the designed SPEC-VA and SPEC-VB constructs (Supplementary Fig. 1a), which express the sfGFP-LEVLFQGP-SsrA polypeptide and intact HRV 3 C protease under the control of T7 and lac promoters, respectively. Our results showed that cells presented almost no sfGFP fluorescence when bearing the SPEC-VA vector only, while exhibited strong recovered sfGFP fluorescence when carrying both SPEC-VA and SPEC-VB vectors (Fig. 1b). Subsequently, we compared the functionality of Tobacco Etch Virus (TEV) and human Rhinovirus 3 C (HRV 3 C) proteases in SPEC system. Both are virus-derived proteases possessing high proteolytic activity and substrate specificity, with HRV 3 C protease exhibiting better proteolytic efficiency and toleration to low temperature^19^. Four common temperatures (37 °C, 30 °C, 25 °C, and 18 °C) were firstly used for system evaluation, because they are normally used for heterologous protein expression in *E. coli*. Our results showed that HRV 3 C protease presented up to 4 folds stronger sfGFP fluorescence intensity than using TEV protease, especially at low temperatures (Fig. 1c). With HRV 3 C protease, the highest levels of sfGFP fluorescence intensity in *E. coli* at 37 °C, 30 °C, 25 °C, and 18 °C were 2.0 ± 0.1 × 10E4, 1.8 ± 0.1 × 10E4, 1.6 ± 0.1 × 10E4 and 1.7 ± 0.1 × 10E4, which was obtained after 4 h, 6 h, 10 h and 20 h IPTG induction, respectively.

### Identification of an optimized split HRV 3 C protease in SPEC system

**Fig. 2:**
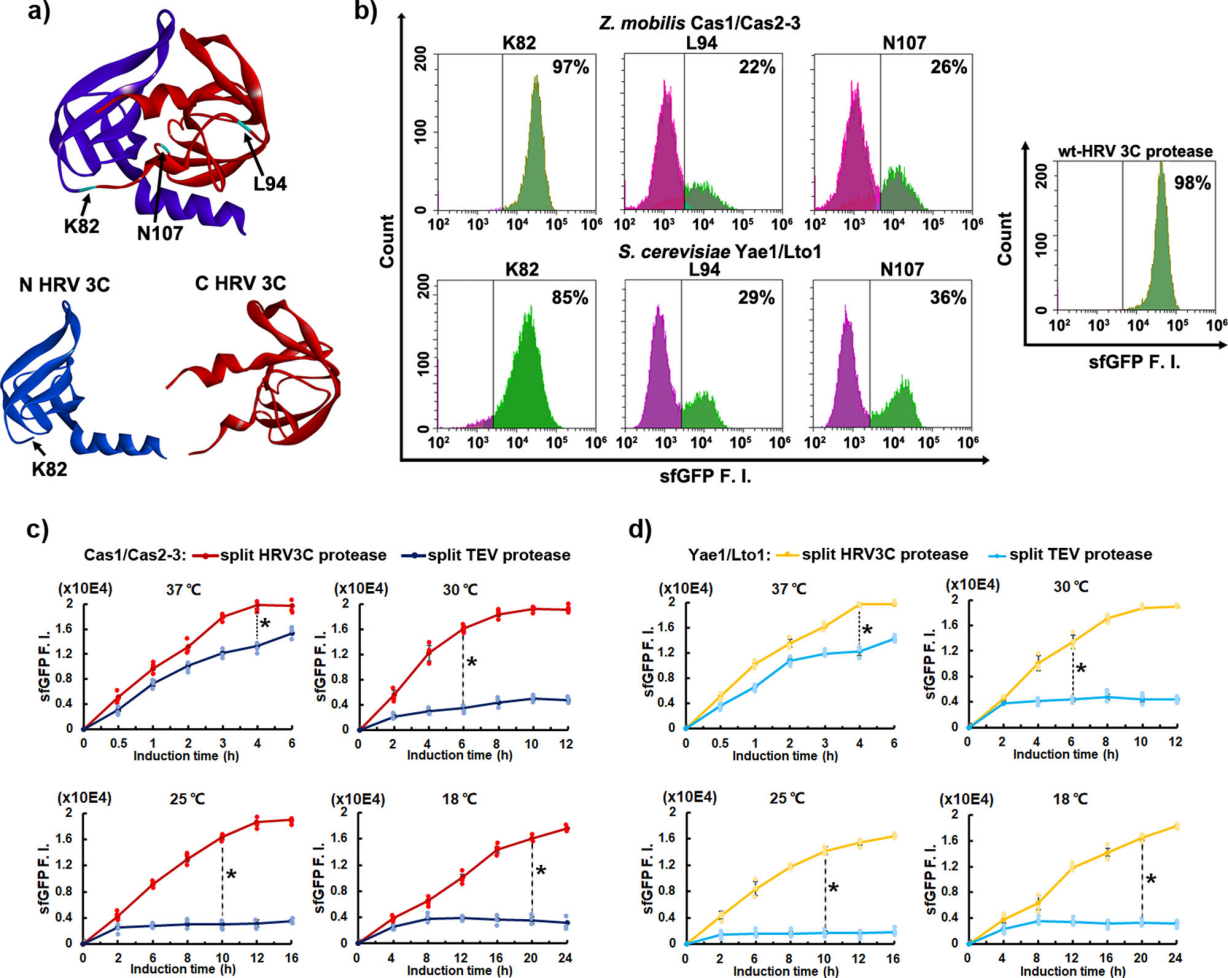
Identification of a highly efficient split HRV 3 C protease and its comparison with split TEV protease in the SPEC system. **a** Designing split HRV 3 C protease based on its structure (PDB: 2B0F) with three split positions (K82, L94, N107). Blue: N-terminal HRV 3 C protease, Red: C-terminal HRV 3 C protease, Purple: the random coil between N-terminal and C-terminal HRV 3 C protease, Cyan: the designed split sites. **b** Characterizing different split HRV 3 C proteases in SPEC system using pSPEC-N107/L94/K82-PP and pSPEC-N107/L94/K82-EP vectors, respectively. Cells bearing pSPEC-VB vector containing full length HRV 3 C protease was used as a positive control (right panel), and cells bearing only pSPEC-VA vector was used as a negative control (Fig. 1b). The total cellular sfGFP F. I. of cells bearing different vectors (pSPEC-K82-EP to pSPEC-N107-PP, Supplementary Table 1) were quantitated through flow cytometry. Upper-left panel, representative flow cytometry histograms of the total cellular sfGFP F.I. of cells expressing different split HRV 3 C proteases fused with *Z. mobilis* Cas1/Cas2-3 protein pair. Lower-left panel, representative flow cytometry histograms of the total cellular sfGFP F.I. of cells expressing different split HRV 3 C proteases fused with *S. cerevisiae* Yae1/Lto1 protein pair. The experiments were performed at 25 °C with 8 h induction. **c** Comparison of split HRV 3 C (K82) protease (Wine line, pSPEC-VC1) with split TEV protease (Dark blue line, pSPEC-VC5) using Cas1/Cas2-3 protein pair in SPEC system at 37 °C, 30 °C, 25 °C, and 18 °C, respectively. **d** Comparison of split HRV 3 C (K82) protease (Yellow line, pSPEC-VC2) with split TEV protease (Cyan line, pSPEC-VC6) using Yae1/Lto1 protein pair in SPEC system at 37 °C, 30 °C, 25 °C, and 18 °C, respectively. All the vector information can be found in Supplementary Table 1. Data are presented as mean ± SEM (*n* = 3 independent experiments) with Student’s t test being performed, ^#^*P* > 0.05, *P ≤ 0.05.

Next, we identified the split HRV 3 C protease modules for SPEC system (Fig. 2a, b). The solved HRV 3 C protease structure indicated that it was constituted of two characteristic six-stranded β-barrel subdomains, connected by a random coil^20^. This structure is similar to the structure of TEV protease, which can also be dissected into two characteristic N-terminal and C-terminal subdomains. The reported split TEV protease modules have the split sites located on the random coil connected N-terminal and C-terminal subdomains^13^. Therefore, K82, L94, and N107, which were located on the random coil and the following β-sheet of the structure of HRV 3 C protease, were selected for generating the split HRV 3 C protease modules. At the same time, two protein pairs, Cas1/Cas2-3 from Type I-F CRISPR/Cas complex of prokaryotic *Zymomonas mobilis*^21^ and Yae1/Lto1 from Fe-S protein assembly (CIA) machinery of eukaryotic *Saccharomyces cerevisiae*^22^, were used to evaluate the effectiveness of split HRV 3 C protease and generality of the SPEC system (Fig. 2b, Supplementary Fig. 1b, Supplementary Fig. 1c). All three split-sites could prompt a re-assembled functional HRV 3 C protease, with K82 being the best. A total of 85–97% cells expressing split HRV 3 C (K82) protease showed sfGFP fluorescence, which is close to the 98% cells expressing the HRV 3 C protease (Fig. 2b). In comparison, only ~40% activity of the TEV protease could be recovered with the reported split TEV protease in a previous report^13^. Moreover, split HRV 3 C (K82) protease outperformed split TEV protease in SPEC system under different temperatures (Fig. 2c, d). It is also worth noting that the highest level of cellular sfGFP fluorescence intensity with re-assembled split HRV 3 C (K82) protease at 30 °C, 25 °C, or 18 °C, was more than 4 folds higher than that of the re-assembled split TEV protease (Fig. 2c, d).

### Construction of the SPEC system in the recombinant *E. coli* BL21 (DE3)-SPEC cells

**Fig. 3:**
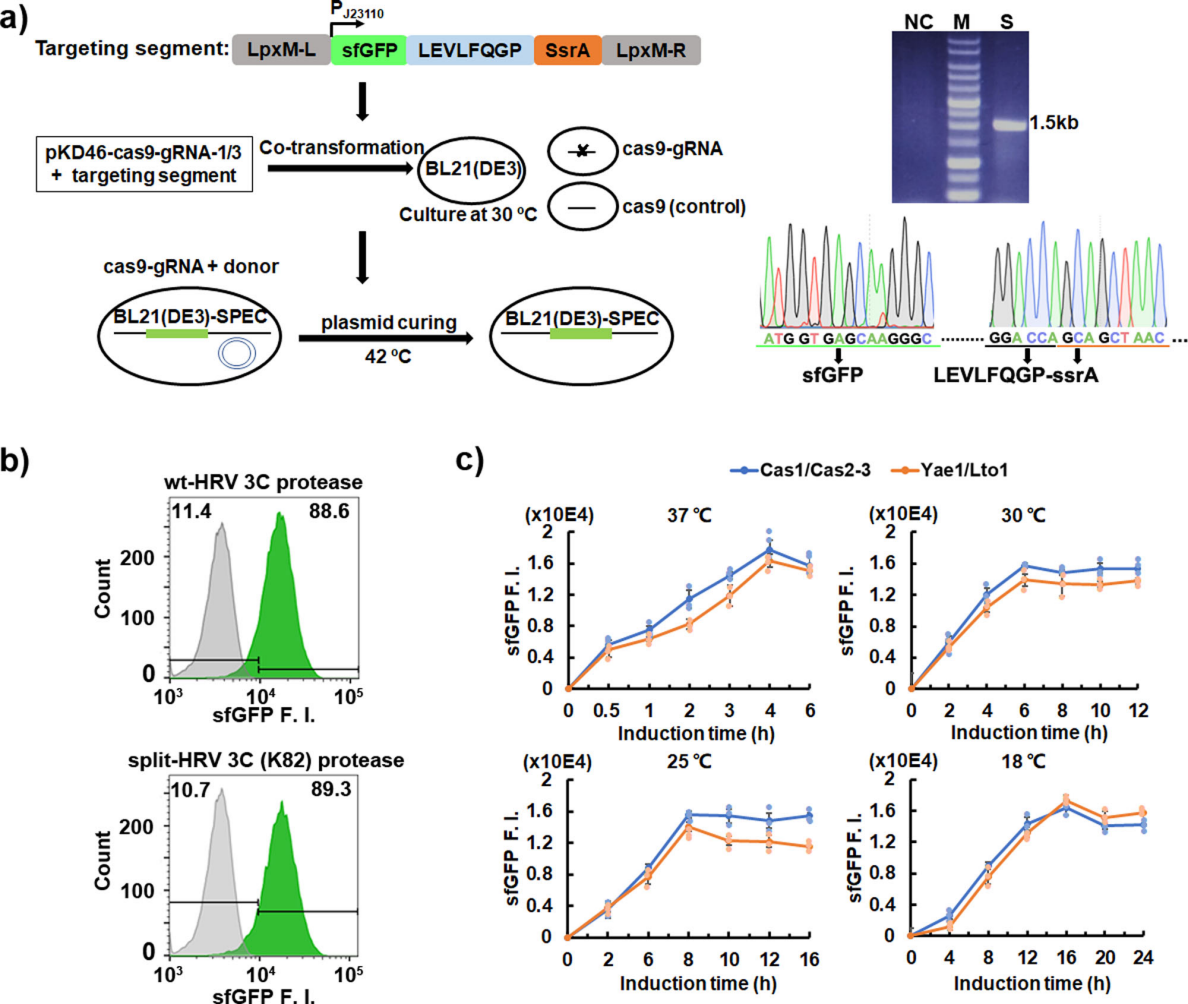
Development of SPEC system in the engineered BL21(DE3)-SPEC cells. **a** Generation of the BL21(DE3)-SPEC cells using no-SCAR incorporated with temperature sensitive plasmid curing strategy. Left panel, scheme of experimental procedure. The *lpxM* gene fragment in the genome of BL21(DE3) strain is replaced by sfGFP-LEVLFQGP-SsrA fragment using CRISPR/Cas9 assisted λ-Red recombinase technology. The pKD46 based vector is used to prompt the temperature sensitive plasmid curing. Upper-right panel, PCR validation of the inserted sfGFP-LEVLFQGP-SsrA fragment in BL21(DE3)-SPEC strain. Negative control (NC): wild-type BL21(DE3) strain. S: BL21(DE3)-SPEC strain. M: 1Kb DNA ladder. Lower-right panel, Sanger sequencing of BL21(DE3) genome to verify the insertion of the sfGFP-LEVLFQGP-SsrA fragment. **b** Validation of the SPEC system in the BL21(DE3)-SPEC strain by comparing the HRV 3 C protease (pSPEC-VB) and split HRV 3 C (K82) protease (pSPEC-VC1) with Cas1/Cas2-3 protein pair. The experiments were performed at 25 °C with 8 h induction. **c** Characterizing split HRV 3 C (K82) protease in BL21(DE3)-SPEC strain with *Z. mobilis* Cas1/Cas2-3 (pSPEC-VC1) and *S. cerevisiae* Yae1/Lto1 (pSPEC-VC2) protein pairs at 37 °C, 30 °C, 25 °C, and 18 °C, respectively. Data are presented as mean ± SEM (*n* = 3 independent experiments). BL21(DE3)-SPEC cells bearing pSPEC-VB plasmid containing HRV 3 C protease was used as a positive control, and the total cellular sfGFP F. I. were quantitated through flow cytometry. All the vector information can be found in Supplementary Table 1.

To simplify the manipulation of SPEC system and increase its stability in *E. coli*, we used the modified no-SCAR (Scarless Cas9 Assisted Recombineering) technology^23^ to integrate the *sfGFP-LEVLFQGP-SsrA* into *E. coli* chromosome followed by temperature sensitive plasmid curing^24^, generating the recombinant *E. coli* BL21(DE3)-SPEC strain (Fig. 3a). Additionally, the prey protein fused N-terminal split HRV 3 C (K82) protease and bait protein fused C-terminal split HRV 3 C (K82) protease were separated into SPEV-VD (Kan^R^) and SPEC-VE (Amp^R^) vectors, respectively (Supplementary Fig. 1d). Our results showed a very low sfGFP background fluorescence in BL21(DE3)-SPEC cells, while cells bearing the SPEC-VB vector exhibited a high sfGFP fluorescence intensity (Fig. 3b). Similar high sfGFP fluorescence intensity was also obtained in BL21(DE3)-SPEC bearing both SPEC-VD and SPEC-VE vectors, validating the effectiveness of this system constituting of BL21(DE3)-SPEC strain and SPEC-VD/SPEC-VE vectors. Subsequently, we evaluated this final SPEC system using Cas1/Cas2-3 and Yae1/Lto1 protein pairs in BL21(DE3)-SPEC at different temperatures (Fig. 3c). Similar tendencies were obtained that split HRV 3 C (K82) protease conferred a higher sensitivity than that of split TEV protease, especially under low temperatures (Fig. 2c, d).

### Characterization of interaction of different effectors in the type I-E and I-F CRISPR/Cas complex

**Fig. 4:**
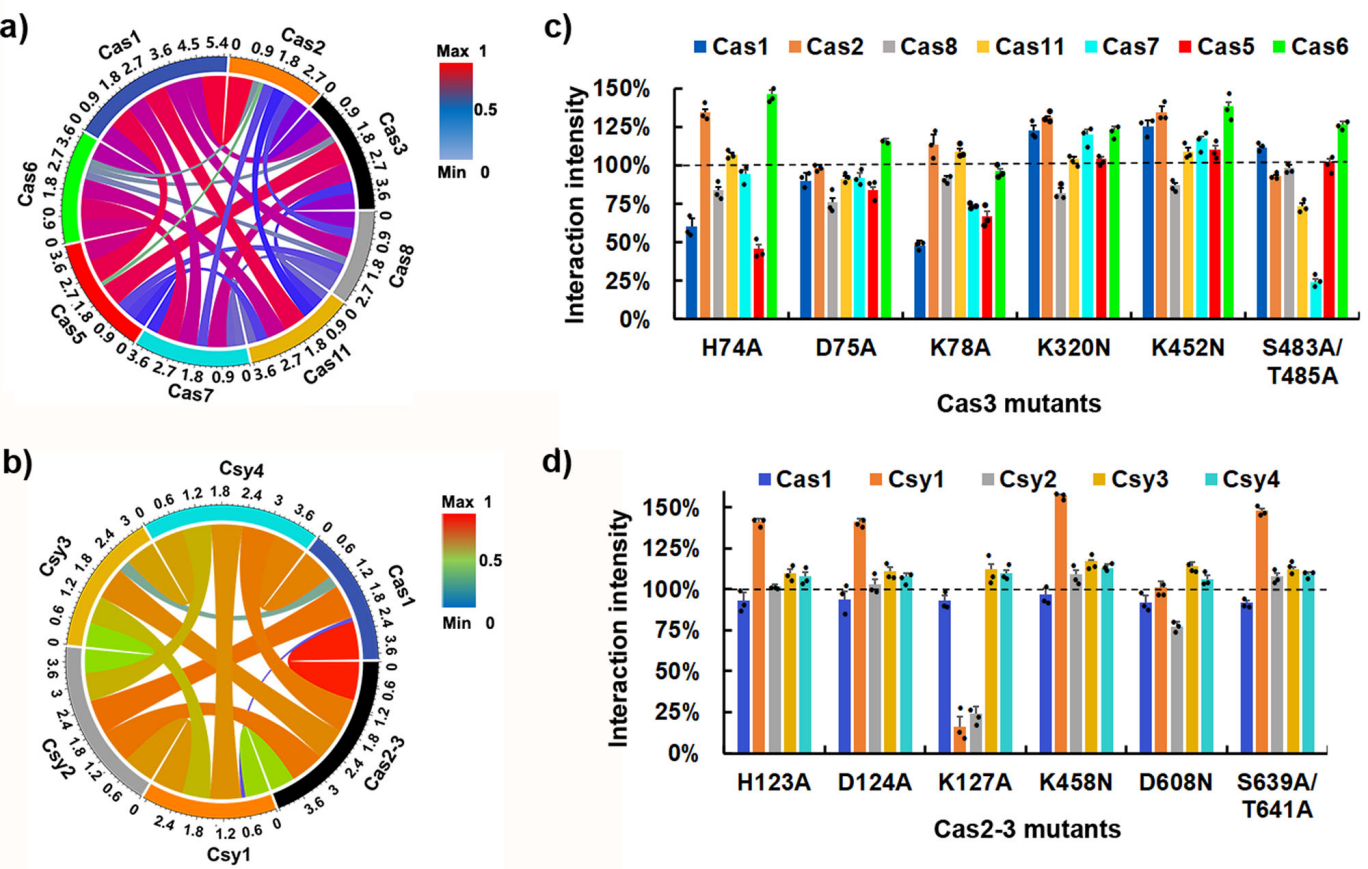
Characterization of protein–protein interactions of effectors in the Type I-E and I-F CRISPR/Cas complex. **a** Protein–protein interactions among eight effectors (Cas1, Cas2, Cas3, Cas8, Cas11, Cas7, Cas5, and Cas6) of *E. coli* K12 Type I-E CRISPR/Cas complex was interpreted using the circular visualization R program^44^. **b** Protein-protein interactions among six effectors (Cas1, Cas2-3, Csy1, Csy2, Csy3, and Csy4) of *Z. mobilis* Type I-F CRISPR/Cas complex was interpreted using the circular visualization R program^44^. **c** The histogram of protein-protein interaction between Cas3 mutants (H74A, D75A, K78A, K320N, D452N, and S483A/T485A, respectively) and seven other effectors (Cas1, Cas2, Cas5, Cas6, Cas7, Cas8, and Cas11) of *E. coli* K12 Type I-E CRISPR/Cas complex. Cas1/GST was used as a negative control (Supplementary Fig. 2b), and Cas1/Cas2 was used as a positive control. Percentage numbers presented correspondent interaction intensities of protein pairs, which were normalized by the interaction between Cas3 and correspondent effectors (Supplementary Fig. 2b). **d** The histogram of protein-protein interaction between Cas2-3 mutants (H123A, D124A, K127A, K458N, D608N, and S639A/T641A, respectively) and five other effectors (Cas1, Csy1, Csy2, Csy3, and Csy4) of *Z. mobilis* Type I-F CRISPR/Cas complex. Cas1/GST was used as a negative control (Supplementary Fig. 3b), and Cas1/Cas2-3 was used as a positive control. Percentage numbers presented correspondent interaction intensities of protein pairs, which were normalized by the interaction between Cas2-3 and correspondent effectors (Supplementary Fig. 3b). All the vector information can be found in Supplementary Table 1. Data are presented as mean ± SEM (*n* = 3 independent experiments).

CRISPR/Cas system is a newly emerged gene-editing tool, in which different effectors work in a corporative manner^25^. We characterized the effector interactions of the type I-E CRISPR/Cas complex from *E. coli* K12 using SPEC system (Fig. 4a, Supplementary Fig. 2). Cas1 and Cas2 exhibited very strong interactions (92%), which is consistent with the reported structure of Cas1-Cas2 complex^26^. Cas1/Cas2, Cas1/Cas5, Cas1/Cas11, Cas3/Cas5, and Cas6/Cas7 presented strong interactions above 80%, and Cas2/Cas5, Cas2/Cas6, Cas3/Cas6, and Cas5/Cas8 exhibited weak interactions. Interestingly, interaction between Cas3 and Cas2 enhanced 32% and 28% with co-overexpression of Cas1 and Cas8, respectively (Supplementary Fig. 2), confirming that the assembly of Cas3 could be prompted by Cas1-Cas2 complex and Cas8 mediated Cascade/R-loop/Cas3 complex^27^. For a comparison, the effector interactions of the type I-F CRISPR/Cas system from *Z. mobilis* were also profiled using the SPEC system (Fig. 4b, Supplementary Fig. 3). Type I-F CRISPR/Cas system is the smallest in Class 1 CRISPR system, containing a characteristic fused Cas2-3 subunit^28^. Similar to type I-E CRISPR/Cas system, a very strong interaction also exists between Cas1 and Cas2-3 (99%), consistent with the reported structure of Cas1_4_-Cas2-3_2_ complex^29^. Differently, most effectors had interaction intensities higher than 60%, among which the interaction intensity of Cas1/Cas2-3, Cas1/Csy2, Cas1/Csy4, Cas2-3/Csy2, Cas2-3/Csy3, Cas2-3/Csy4, Csy1/Csy2, and Csy1/Csy4 were above 80%. However, Cas1 presented very weak interaction with Csy3 (14%) and no interaction with Csy1 (0%), indicating a characteristic type I-F CRISPR/Cas complex structure.

To further understand the difference between type I-E and I-F CRISPR/Cas complexes, we profiled the interactions of Cas3 mutants^30^ with all other effectors (Fig. 4c, d). Surprisingly, the Cas3 mutants presented different effects in these two complexes. In type I-E CRISPR/Cas complex, most Cas3 mutants increased its interaction with Cas2 and Cas6, while mutations altering Mg^2+^-dependent HD-nuclease activity (H74A, D75A, K78A) and ATP-dependent helicase activity (K320N, K452N, S483A/T485A) either decreased or increased its interaction with Cas1, respectively. In comparison, most Cas3 mutants did not affects the interaction of Cas2-3 with other effectors in type I-F CRISPR/Cas complex. However, K127A significantly decreased the interaction of Cas2-3 with Csy1 (85%) and Csy2 (75%), while other mutants largely increased its interaction with Csy1 (>40%). These results indicated different roles of Cas3 in type I-E and I-F CRISPR/Cas systems, in which Cas3 might possess a more stable structure due to its fusion with Cas2 effector. Besides, this difference on effector interaction might lead to the different functional mechanisms between these two CRISPR/Cas systems.

## Discussion

In our studies here, a designed split HRV 3 C protease was utilized in the SPEC system for quantitating protein-protein interaction from both prokaryotic and eukaryotic species. Compared to the widely used split TEV protease^13,31^, HRV 3 C protease is catalytically faster, thus producing further enhanced sfGFP signals in SPEC system (Fig. 1c). More importantly, HRV 3 C protease exhibit constantly high proteolytic activity in a broad temperature range from 18 °C to 37 °C (Fig. 1c). This property is very important because many heterologous proteins could only be expressed and folded well at low temperature in *E. coli* cells. Besides the common temperatures for *E. coli* cell growth, we also evaluated the SPEC system at 42 °C and 12 °C. Similar results were obtained that split HRV 3 C (K82) protease presented higher sensitivity than that of split TEV protease at 12 °C, while their difference is not obvious at 42 °C (Supplementary Fig. 4). These results further confirmed that split HRV 3 C (K82) protease possesses high sensitivity in a broad temperature range, which is better than the split TEV protease, especially at low temperature. Besides the advantageous adaption to low temperature, it is also noticed that the reassembled split HRV 3 C proteases presented almost the identical proteolytical activity to the intact HRV 3 C protease (Fig. 2b), while it was reported that reassembled split TEV protease only recovers its 40% proteolytic activity^13^. The high activity and low-temperature adaptability of split HRV 3 C (K82) protease make it more efficient in SPEC system.

SPEC systems have some unique advantages. Comparing to split GFP method, the split HRV 3 C protease can amplify the signals of PPIs, thus detecting the weak interactions. Some other PCAs can also amplify the signals of PPIs, such as split luciferase assay^32^. However, SPEC system has another advantage that it quantitates the PPIs through fluorescent intensity of cellular sfGFP, not requiring additional exogenous substrates. It also needs to point out that SPEC system also has limitations. Since SPEC system is an *E. coli* cell-based assay, it requires that both the prey and bait proteins can be expressed and functionally folded in *E. coli* cells. Therefore, it may not be suitable for complex eukaryotic proteins that cannot be folded in *E. coli* cells.

To stabilize the production of sfGFP in *E. coli* cells, we integrated the sfGFP gene into the *E. coli* genome in SPEC, which could minimize the heterogeneity caused by episomal plasmid (Fig. 3). The modular elements make the SPEC system highly flexible. Using other reporters, SPEC system may be performed with preferred signal readout. Additionally, owing to the sensitivity and flexibility of split HRV3C protease, we believe that it could be used in the future to design more useful biosensors, in particular for high-throughput applications requiring signal enhancement. In addition, SPEC system uses a two-plasmid strategy in the recombinant *E. coli* BL21(DE3)-SPEC strain. The separation of prey and bait proteins in different plasmids not only significantly simplifies the manipulation, but also facilitates the analysis of large-scale PPIs. Combined with the flow cytometry and high-throughput sequencing technology, it could be believed that SPEC system can be extended to characterize PPIs in a library against library set.

**Fig. 5:**
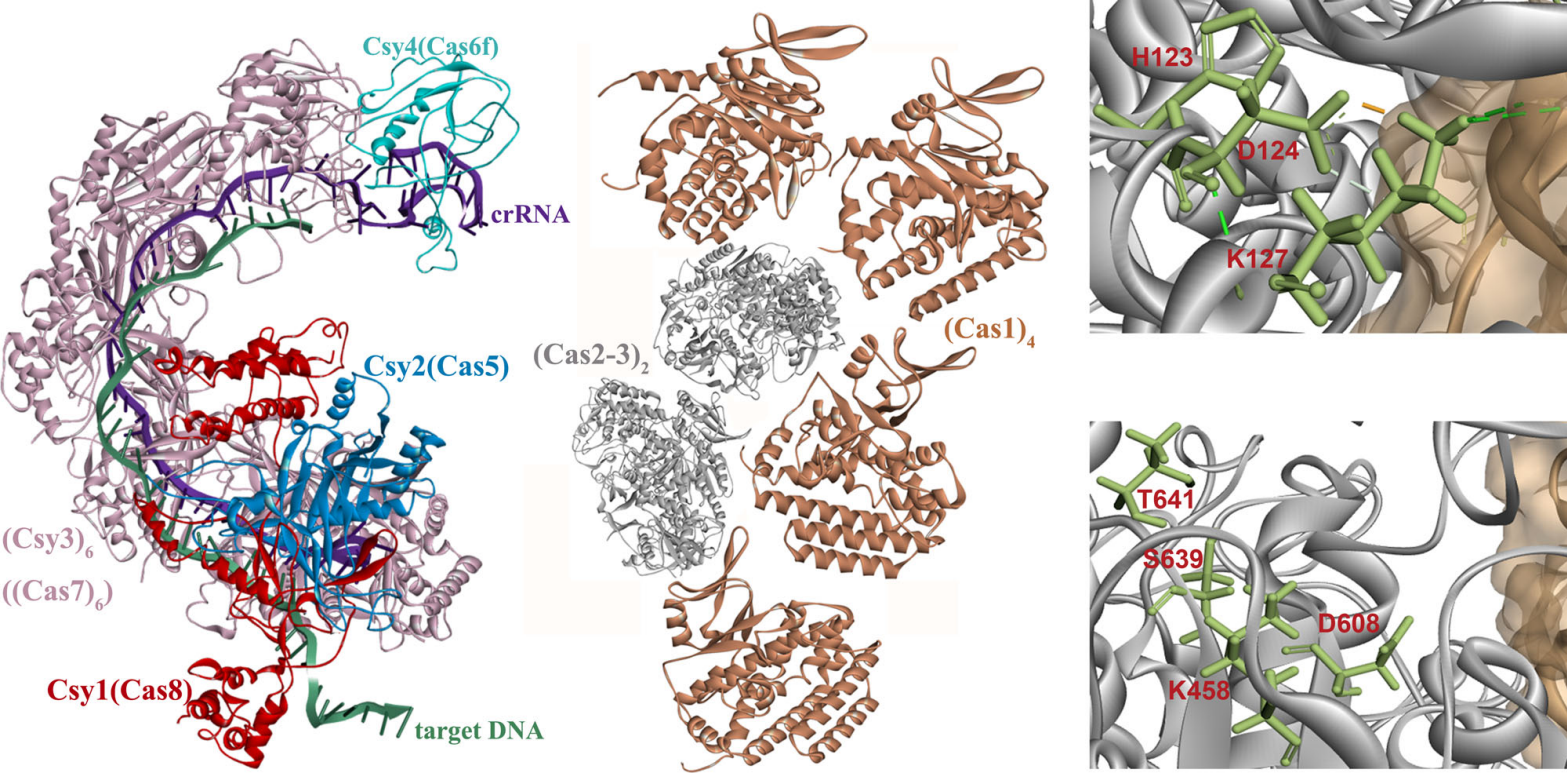
Simulated structure diagram of Type I-F CRISPR/Cas complex. Left panel: Model protein structures of six proteins (Cas1, Cas2-3, Csy1, Csy2, Csy3 and Csy4) in CRISPR/Cas complex. Right panel: Location of mutation sites (H123, D124, K127, K458, D608, S639, and T641; red font) in Cas2-3.

Combining our PPIs results with the previous reported structures of Cas1_4_–Cas2-3_2_ complex^29^ and resembled type I-E CRISPR/Cas complex^26^, we simulated the model structure scheme of type I-F CRISPR/Cas complex (Fig. 5). Our results showed that Cas2-3 presented strong interactions with all other effectors, and Cas1 presented a very strong interaction with Cas2-3, Csy2, and Csy4, but no interaction of Csy1 and very low interaction with Cys3 (Fig. 4b, Supplementary Fig. 3). We speculated that Cas1 effector is located outside of the whole complex, wrapping up the Cas2-3 effectors, with Csy1 and Csy3 effectors being located at the other side of the whole complex. This unique structure might be caused by the requirement of the “flexible” PAM recognition function of Csy1^33^, and the binding of the spacer region by Csy3 in type I-F CRISPR systems^34^. Additionally, our results also confirmed that Cas2 might function as adaption protein and the helicase and nuclease functions of the Cas3 domain were involved in interference process^35^.

The simulated type I-F CRISPR/Cas complex structure (Fig. 5) exhibits high similarity with that of the type I-E cascade complex^26^. Interestingly, Cas2 and Cas3 only presented an interaction of 56%, while this interaction was significantly enhanced to 90% and 86% when co-expression of Cas1 and Cas8 (Fig. 4a, Supplementary Fig. 2), respectively. This might explain the characteristically fused Cas2-Cas3 domain in type I-F CRISPR/Cas complex, because it lacks Cas8 effector. Cas3 is the catalytic effector in type I CRISPR/Cas systems, and the fused Cas2-Cas3 domain is believed to maintain a more stable function of Cas3 in type I-F CRISPR/Cas complex, which is further supported in our studies of Cas3 mutants in both type I-E and I-F CRISPR/Cas complexes (Fig. 4c, d). Comparing to the Cas3 mutants in type I-E CRISPR/Cas complex, which exhibited promiscuous effect, the correspondent mutations in type I-F CRISPR/Cas complex all presented similar interactions with all other effectors, except the nuclease activity related Cas2-3 (K127A) mutant presented obviously decreased interaction with Csy1/Csy2 (from 63% to 10%, 88% to 21%, respectively). Besides, Cas2-3(H123A, D124A, K458N, and S639A/T641A) presented a significantly enhanced interaction with Csy1. All these Cas3 related mutations affect the interactions of Cas2-Cas3 with Csy1/Csy2 effectors, which were located at N-terminus of crRNA-Cascade complex, and speculated to function as blocking nuclease recruitment, crRNA and target binding^36^.

In principle, any protein pairs that are able to be functionally expressed in *E. coli* cells at the temperature range from 12 °C and 42 °C. can be characterized in SPEC system. Considering the exist of similar ClpXP-SsrA mediated protein degradation machinery in other bacteria, such as the Gram-positive model species *Bacillus subtills*^37^, the concept of SPEC system can be easily implanted in other bacteria.

## Methods

### Bacterial strains and reagents

*E. coli* XL10-Gold strain (Stratagene, San Diego, USA) was used for plasmid construction, and *E. coli* BL21(DE3) strain was used for protein expression and flow cytometry analysis. Culture media, chemicals, and restriction enzymes were purchased from BD Biosciences (San Jose, CA, USA), Sigma-Aldrich (St. Louis, MO, USA), and New England Biolabs (Ipswich, MA, USA), respectively.

### Plasmid construction

pET28a and pUC19 plasmids were used as backbone to construct pSPEC plasmids in this study (Supplementary Fig. 1). Total of 64 constructs were generated and their vector information can be found in Supplementary Table 1. The split HRV 3 C protease domain (N HRV 3 C or C HRV 3 C) was fused with different protein pairs (prey and bait proteins), forming a prey-N HRV 3 C protease or bait-C HRV 3 C protease cassette. HRV 3C protease substrate sequence was flanked with sfGFP and SsrA tag (AANDENYALAA) sequences, forming the sfGFP-LEVLFQGP-AANDENYALAA cassette. A well-characterized Tobacco Etch Virus (TEV) protease variant containing a S219P mutation with enhanced stability^38^ was used and annotated as TEV Protease in our research. Similarly, TEV protease substrate sequence (ENLYFQS) was also flanked with sfGFP and SsrA tag sequences, forming the sfGFP- ENLYFQS-AANDENYALAA cassette. These cassettes were cloned into different pSPEC plasmids.

Plasmids for this study was constructed using T5 exonuclease DNA assembly (TEDA) assay with minor modification^39^. Firstly, 1 mL 5 × TEDA solution contained 0.5 M Tris-HCl pH7.5, 50 mM dithiothreitol, 0.25 g of PEG-8000, and 1 μL of 10 U/μL T5 exonuclease (New England Biolabs, USA) was prepared for cloning. For the experiment, 4 μL assembly mixture aliquot was thawed on ice, followed by adding 16 μL of DNA solution containing the linear vector and target inserts. The molar ratio of linearized vector to target insert was set as 1:3, with vector amount approximately 50 to 200 ng. Reactions were then performed at 30 °C for 40 min, followed by immediate transformation into *E. coli* competent cells.

### Knock-in of *sfGFP-LEVLFQGP*-*SsrA* fragment into the genome of *E. coli*

*E. coli* BL21(DE3) (*lpxM*:: *sfGFP-LEVLFQGP-SsrA*) strain, named as BL21 (DE3)-SPEC, was generated using CRISPR/Cas9 assisted λ-Red recombinase system^40^ followed by temperature sensitive plasmid curing. Cas9 and gRNA were incorporated into pKD46, a temperature-sensitive vector^41^ carrying λ-Red genes (γ, β, exo), which promotes recombination, to construct pKD46-cas9-gRNA plasmid (Supplementary Fig. 1). Subsequently, pKD46-cas9-gRNA vector DNAs targeting the *lpxM* gene in *E. coli* genome were transformed into *E. coli* BL21(DE3) followed by cultivation and induction with 0.2% L-arabinose in LB media containing 100 μg/mL ampicillin at 30 °C to an OD600 of approximately 0.6. The electrocompetent cells were then made by centrifugation, followed by washing twice with ice-cold deionized water. Meanwhile, the donor fragment (sfGFP-LEVLFQGP-SsrA) was gel-purified, and 10 ng of donor DNA and 50 μL of electrocompetent cells were then mixed on ice for electroporation. One mL LB media were then added into the post-shocked cells immediately following by recovering at 37 °C for 1 h, and then spreading on LB plates with chloramphenicol. *E. coli* transformants carrying sfGFP-LEVLFQGP-SsrA fragments in the genome were screened and selected by colony PCR and Sanger sequencing. The validated transformants were then grown in LB media at 42 °C for 12 h to eliminate the temperature-sensitive pKD46-cas9-gRNA-donor plasmid, generating BL21(DE3)-SPEC strain for further experiments.

### Protein-Protein Interaction analysis with CytoFLEX by SPEC assay

The pSPEC constructs were transformed into *E. coli* BL21(DE3) or BL21(DE3)-SPEC cells followed by cultivating at 37 °C until OD_600_ reached 0.8. The cells were then induced by 0.5 mM IPTG (final concentrations) at different temperatures ranging from 18 °C to 37 °C.

Two hundred µL induced cells were collected by centrifugation at 3,000 × *g* at 4 °C for 5 min and then resuspended into 400 µL 1 × PBS buffer (200 mM NaCl, 66 mM Na_2_HPO_4_, 34 mM NaH_2_PO_4_. 2H_2_O, pH 7.4), followed by sfGFP fluorescent analysis using Beckman Coulter CytoFLEX Flow Cytometer (Beckman Coulter, USA) equipped with a 488 nm laser and a 525/40 nm band-pass filter. sfGFP fluorescent intensity (sfGFP F. I.) of the cells was measured by the mean fluorescence intensity of the fluorescent cells.

The protein-protein interaction (PPI) intensity of cells expressing different targeting protein pairs were normalized using the mean fluorescence intensity of the cells expressing wt HRV 3 C protease as a control. The normalized interaction intensity was calculated as: [Normalized interaction intensity] = ([Mean fluorescence intensity percentage of the cells] / [Percentage of whole cells]) × 100%.

### Structure modeling and circular visualization analysis for PPIs data

The structure modeling of six proteins (Cas1, Cas2-3, Csy1, Csy2, Csy3, and Csy4) from *Z. mobilis* Type I-F CRISPR/Cas complex were performed by using the I-TASSER on-line server^42^ respectively. The docking analyses of type I-F CRISPR/Cas complex were performed by using the ZDOCK program in BIOVIA Discovery Studio client 2020^43^. The circular visualization map of protein-protein interactions among six proteins (Cas1, Cas2-3, Csy1, Csy2, Csy3, and Csy4) from *Z. mobilis* Type I-F CRISPR/Cas complex was generated by using *circlize* package in R^44^.

### Statistics and reproducibility

sfGFP fluorescent analysis and the protein-protein interaction (PPI) intensity of cells assays were independently replicated three times and individual data points are reported for each experiment.

### Reporting summary

Further information on research design is available in the Nature Research Reporting Summary linked to this article.

## Supplementary information

Supplementary information

Supplementary Data 1

Reporting Summary

Description of Supplementary Files

## Data Availability

The source data for the graphs in the main figures is available as Supplementary Data 1. The vectors in Supplementary Figure 1 have been deposited in to Addgene with ID numbers 171974-171989. The datasets generated and/or analyzed during the current study are available from the authors on reasonable request.
