# Supplementary material for: A split protease-E. coli ClpXP system quantifies protein–protein interactions in Escherichia coli cells
Source: Commun Biol. 2021 Jul 6;4:841. doi: 10.1038/s42003-021-02374-w (PMC8260793; doi:10.1038/s42003-021-02374-w)
Supplement: Supplementary file 1 — Supplementary information [file 42003_2021_2374_MOESM1_ESM.pdf]

# A split protease-*E. coli* ClpXP system quantifies protein-protein interactions in *Escherichia coli* cells

Shengchen Wang[1], Faying Zhang[1], Meng Mei[1], Ting Wang[1], Yueli Yun[1], Shihui Yang[1], Guimin Zhang[1]*, Li Yi[1]*

[1] State Key Laboratory of Biocatalysis and Enzyme Engineering, School of Life Sciences, Hubei University, Hubei, China

*To whom correspondence should be addressed:
Li Yi, Ph.D
Professor, Department of Bioengineering, Department of Microbiology
State Key Laboratory of Biocatalysis and Enzyme Engineering
School of Life Sciences, Hubei University
368 Youyi Road, Wuchang District, Wuhan,
Hubei, China 430062
Tel.: +86-27-88661237; Fax: +86-27-88663882
Email: liyi@hubu.edu.cn

Guimin Zhang, Ph.D.
Professor, Department of Microbiology
State Key Laboratory of Biocatalysis and Enzyme Engineering
School of Life Sciences, Hubei University
368 Youyi Road, Wuchang District, Wuhan,
Hubei, China 430062,
Tel.: +86 27 88661746 Fax: +86 27 88663882
E-mail: zhangguimin6@hotmail.com

**Supplementary Table**

**Supplementary Table 1. Constructs generated in this work**

| Construct | Description | Source |
|---|---|---|
| pSPEC | pUC19 with expression unit of pET28a | This study |
| pSPEC-T | pSPEC with TEV protease | This study |
| pSPEC-VA | pET28a with sfGFP-LEVLFQGP-SsrA | This study |
| pSPEC-VB | pSPEC with HRV3C protease | This study |
| pSPEC-N107-PP | split site: G1- N107; with Cas1/Cas2-3 | This study |
| pSPEC-L94-PP | split site: G1-L94; with Cas1/Cas2-3 | This study |
| pSPEC-K82-PP | split site: G1-K82; with Cas1/Cas2-3 | This study |
| pSPEC-N107-EP | split site: G1- N107; with Yae1/Lto1 | This study |
| pSPEC-L94-EP | split site: G1- L94; with Yae1/Lto1 | This study |
| pSPEC-K82-EP | split site: G1- K82; with Yae1/Lto1 | This study |
| pSPEC-VC1 | Cas1-NHRV 3C protease--Cas2-3-CHRV 3C protease | This study |
| pSPEC-VC2 | Yae1-NHRV 3C protease--Lto1-CHRV 3C protease | This study |
| pSPEC -VC3 | Cas1-NHRV 3C protease--GST-CHRV 3C protease | This study |
| pSPEC-VC4 | Rli1-NHRV 3C protease--Lto1-CHRV 3C protease | This study |
| pSPEC-VC5 | Cas1-NTEV protease--Cas2-3-CTEV protease | This study |
| pSPEC-VC6 | Yae1-NTEV protease--Lto1-CTEV protease | This study |
| pSPEC-VD | pET28a- prey- NHRV 3C protease | This study |
| pSPEC-VE | pSPEC- bait- CHRV 3C protease | This study |
| pSPEC-VD1 | Cas1-NHRV 3C protease | This study |
| pSPEC-VE1 | Cas2-3-CHRV 3C protease | This study |
| pSPEC-VD2 | Yae1-NHRV 3C protease | This study |
| pSPEC-VE2 | Lto1-CHRV 3C protease | This study |
| pSPEC-VD3 | Csy1-NHRV3C protease | This study |
| pSPEC-VD4 | Csy2-NHRV3C protease | This study |
| pSPEC-VD5 | Csy3-NHRV3C protease | This study |
| pSPEC-VD6 | Csy4-NHRV3C protease | This study |
| pSPEC-VE3 | Csy1-CHRV3C protease | This study |
| pSPEC-VE4 | Csy2-CHRV3C protease | This study |
| pSPEC-VE5 | Csy3-CHRV3C protease | This study |
| pSPEC-VE6 | Csy4-CHRV3C protease | This study |
| pUC19-N | NHRV 3C protease, without prey (*Sac* I and *Pst* I) | This study |
| pUC19-C | CHRV 3C protease, without bait (*Sac* I and *Pst* I) | This study |
| pKD46-Cas9-gRNA-1 | pSC101 *ori*, gRNA with spacer 1 | This study |
| pKD46-Cas9-gRNA-3 | pSC101 *ori*, gRNA with spacer 3 | This study |
| pKD46-Cas9-gRNA-1-donor | *Xho* I, donor: lpxM-L-sfGFP-LEVLFQGP-SsrA-lpxM-R | This study |
| pKD46-Cas9-gRNA-3-donor | *Xho* I, donor: lpxM-L-sfGFP-LEVLFQGP-SsrA-lpxM-R | This study |
| pSPEC-VD7 | K12_Cas1-NHRV 3C protease | This study |
| pSPEC-VD8 | K12_Cas2-NHRV 3C protease | This study |
| pSPEC-VD9 | K12_Cas3-NHRV 3C protease | This study |

| | | |
|---|---|---|
| pSPEC-VD10 | K12_Cas5-NHRV 3C protease | This study |
| pSPEC-VD11 | K12_Cas6-NHRV 3C protease | This study |
| pSPEC-VD12 | K12_Cas7-NHRV 3C protease | This study |
| pSPEC-VD13 | K12_Cas8-NHRV 3C protease | This study |
| pSPEC-VD14 | K12_Cas11-NHRV 3C protease | This study |
| pSPEC-VE7 | K12_Cas1-CHRV 3C protease | This study |
| pSPEC-VE8 | K12_Cas2-CHRV 3C protease | This study |
| pSPEC-VE9 | K12_Cas3-CHRV 3C protease | This study |
| pSPEC-VE10 | K12_Cas5-CHRV 3C protease | This study |
| pSPEC-VE11 | K12_Cas6-CHRV 3C protease | This study |
| pSPEC-VE12 | K12_Cas7-CHRV 3C protease | This study |
| pSPEC-VE13 | K12_Cas8-CHRV 3C protease | This study |
| pSPEC-VE14 | K12_Cas11-CHRV 3C protease | This study |
| pSPEC-VE15 | Cas2-3(H123A)-CHRV 3C protease | This study |
| pSPEC-VE16 | Cas2-3(D124A)-CHRV 3C protease | This study |
| pSPEC-VE17 | Cas2-3(K127A)-CHRV 3C protease | This study |
| pSPEC-VE18 | Cas2-3(K458N)-CHRV 3C protease | This study |
| pSPEC-VE19 | Cas2-3(D608N)-CHRV 3C protease | This study |
| pSPEC-VE20 | Cas2-3(S639A/T641A)-CHRV 3C protease | This study |
| pSPEC-VE21 | Cas3(H74A)-CHRV 3C protease | This study |
| pSPEC-VE22 | Cas3(D75A)-CHRV 3C protease | This study |
| pSPEC-VE23 | Cas3(K78A)-CHRV 3C protease | This study |
| pSPEC-VE24 | Cas3(K320N)-CHRV 3C protease | This study |
| pSPEC-VE25 | Cas3(K452N)-CHRV 3C protease | This study |
| pSPEC-VE26 | Cas3(S639A/T641A)-CHRV 3C protease | This study |

# Supplementary Figures

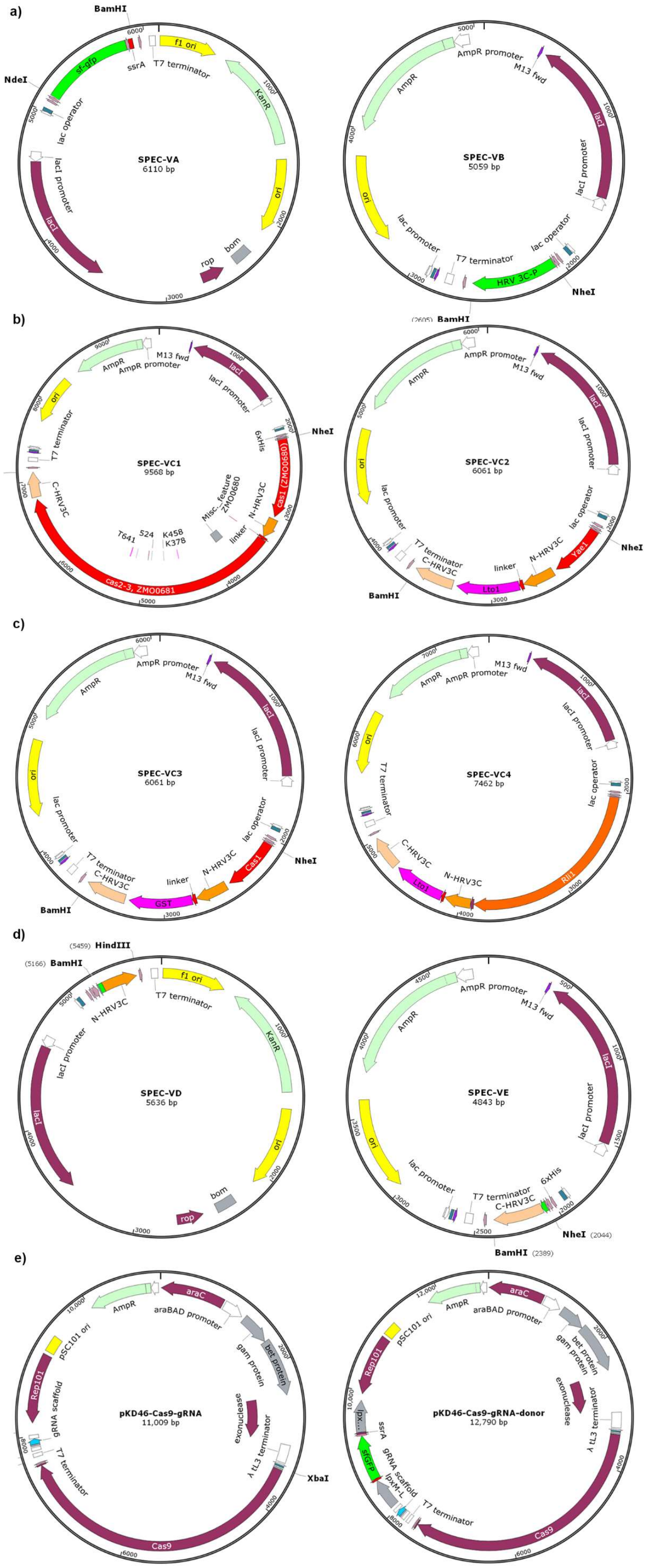


**Supplementary Figure 1 Plasmid maps of SPEC system vectors.**

**a** Plasmid maps of pSPEC-VA and pSPEC-VB, which were used for expression of sfGFP-LEVLFQGP-SsrA and HRV 3C protease, respectively. **b** Plasmid maps of pSPEC-VC1 and pSPEC-VC2, which were used for expression of Cas1-NHRV 3C protease--Cas2-3-CHRV 3C protease cassette and Yae1-NHRV 3C protease--Lto1-CHRV 3C protease cassette, respectively. **c** Plasmid maps of pSPEC-VC3 and pSPEC-VC4, which were used for expression of Cas1-NHRV 3C protease--GST-CHRV 3C protease cassette and Rli1-NHRV 3C protease--Lto1-CHRV 3C protease cassette, respectively. **d** Plasmid maps of pSPEC-VD and pSPEC-VE, which were used for expression of prey-NHRV 3C protease fused protein and bait-CHRV 3C protease fused protein, respectively. **e** Plasmid maps of pKD46-Cas9-gRNA and pKD46-Cas9-gRNA-donor, which were used for construction of BL21(DE3)-SPEC recombination strain.

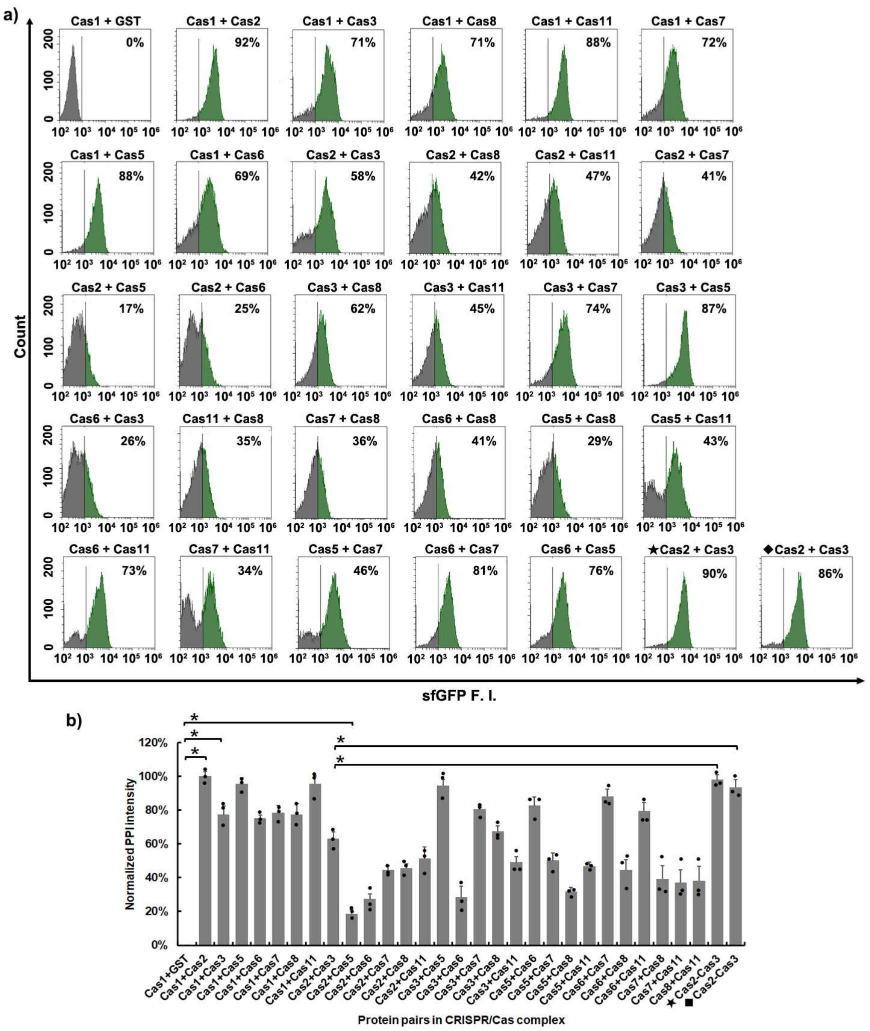


**Supplementary Figure 2 PPIs of effectors in the Type I-E CRISPR/Cas complex quantified by SPEC system**

**a** Representative flow cytometry results of the PPIs of effectors in the Type I-E CRISPR/Cas complex. The total cellular sfGFP fluorescence intensity (sfGFP F. I.) of cells bearing different cassettes were quantified using flow cytometry (detected with the FITC channel, 525/40 nm BP). **b** The statistical analysis of cellular sfGFP F. I. of *E. coli* bearing different interacted protein pairs from Type I-E CRISPR/Cas complex. Cas1/GST protein pair was used as a negative control. [Normalized interaction intensity] = ([Mean fluorescence intensity percentage of the cells] / [Percentage of whole cells]) × 100%. In these results, Cas1/GST is used as a negative control; Cas1/Cas2 is used as a positive control; ★ Cas2/Cas3 and ◆ Cas2/Cas3 represent that the interaction between Cas2 and Cas3 with co-overexpression of Cas1 and Cas8, respectively. All results were normalized by the interaction intensity of Cas1/Cas2. The vector information can be found in **Supplementary Table 1**. Data are presented as mean ± SEM (n=3 independent experiments) with Student's t test being performed, $^{\#}P > 0.05$, $^{*}P \leq 0.05$.

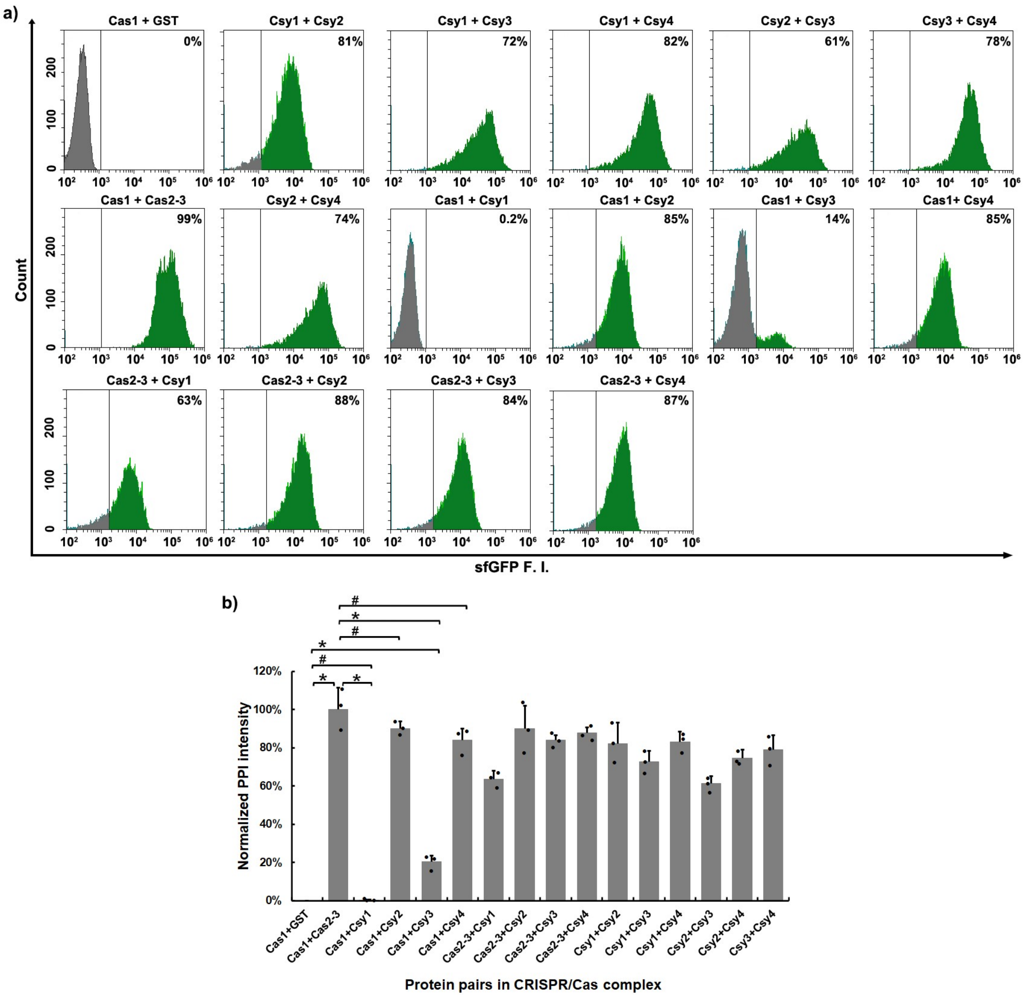


**Supplementary Figure 3 PPIs of effectors in the Type I-F CRISPR/Cas complex quantified by SPEC system**

**a** Representative flow cytometry results of the PPIs of effectors in the Type I-F CRISPR/Cas complex. The total cellular sfGFP fluorescence intensity (sfGFP F. I.) of cells bearing different cassettes were quantified using flow cytometry (detected with the FITC channel, 525/40 nm BP). **b** The statistical analysis of cellular sfGFP F. I. of *E. coli* bearing different interacted protein pairs from Type I-F CRISPR/Cas complex. Cas1/GST protein pair was used as a negative control. [Normalized interaction intensity] = ([Mean fluorescence intensity percentage of the cells] / [Percentage of whole cells]) × 100%. In these results, Cas1/GST is used as a negative control; Cas1/Cas2-3 is used as a positive control. All results were normalized by the interaction intensity of Cas1/Cas2-3. The vector information can be found in **Supplementary Table 1**. Data are presented as mean ± SEM (n=3 independent experiments) with Student's t test being performed, $^{\#}P > 0.05$, $^{*}P \leq 0.05$.

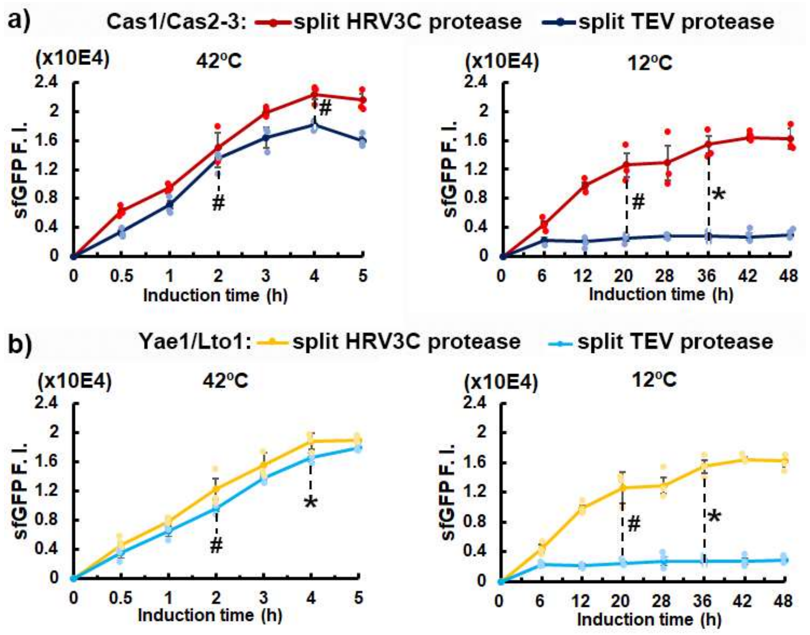


**Supplementary Figure 4 Comparison of split HRV 3C (K82) protease with split TEV protease in SPEC system at 42 °C and 12 °C.**

**a** Comparison of split HRV 3C (K82) protease (Wine line, pSPEC-VC1) with split TEV protease (Dark blue line, pSPEC-VC5) using Cas1/Cas2-3 protein pair in SPEC system at 42 °C and 12 °C, respectively. **b** Comparison of split HRV 3C (K82) protease (Yellow line, pSPEC-VC2) with split TEV protease (Cyan line, pSPEC-VC6) using Yae1/Lto1 protein pair in SPEC system at 42 °C and 12 °C, respectively. All the vector information can be found in **Supplementary Table 1**. Data are presented as mean ± SEM (n=3 independent experiments) with Student's t test being performed, $^{\#}P > 0.05$, $^{*}P \leq 0.05$.
