# Supplementary material for: A split protease-E. coli ClpXP system quantifies protein–protein interactions in Escherichia coli cells
Source: Commun Biol. 2021 Jul 6;4:841. doi: 10.1038/s42003-021-02374-w (PMC8260793; doi:10.1038/s42003-021-02374-w)
Supplement: Supplementary file 4 — Description of Supplementary Files [file 42003_2021_2374_MOESM4_ESM.pdf]

Description of Additional Supplementary Files

File name: Supplementary Data 1
Description: Source data for all the graphs and charts in the main figures.
